# Genome-wide characterization of the *HpHsf* gene family and expression analysis under heat stress in *Herpetospermum pedunculosum*

**DOI:** 10.3389/fpls.2025.1701203

**Published:** 2025-12-18

**Authors:** Ziwei Zhu, Haijing Liang, Min Sun, Yixi Yang, Xiaoying Qin, Qi Zhao, Rui Li, Yang Tao

**Affiliations:** 1 Natural Products Chem-bio Innovation Center, Chengdu University, Chengdu, China; 2 Engineering Research Center of Sichuan-Xizang Traditional Medicinal Plant, Chengdu University, Chengdu, China; 3 Institute for Advanced Study, Chengdu University, Chengdu, China; 4 School of Food and Biological Engineering, Chengdu University, Chengdu, China; 5 Sichuan Liangyuan Food Co., Ltd., Bazhong, China

**Keywords:** *Herpetospermum pedunculosum*, heat shock transcription factors, gene family, heat stress, expression profiling

## Abstract

**Introduction:**

Heat shock transcription factors (Hsfs) are key regulators of plant responses to heat stress and other environmental challenges. However, the *Hsf* gene family of *Herpetospermum pedunculosum*, an alpine medicinal plant valued for its hepatoprotective properties, remains poorly characterized. Investigating the characteristics of the *Hsf* genes in *H. pedunculosum* will enhance our understanding of its thermotolerance mechanisms and provide a theoretical basis for improving its environmental adaptability.

**Methods:**

In this study, we conducted a genome-wide identification and characterization of the *Hsf* gene family in *H. pedunculosum*. The study included analyses of protein physicochemical properties, chromosome locations, phylogenetic classification, conserved motifs, gene structures, collinearity, and cis-acting promoter elements. qRT-PCR was performed to assess the transcription levels of *HpHsf* genes in *H. pedunculosum* leaves under heat stress over a time course. Three representative *HpHsfs* were selected for subcellular localization analysis.

**Results:**

Here, a total of 21 *HpHsf* genes were identified. Phylogenetic analysis classified the *HpHsfs* into three main groups (A, B, and C), consistent with classifications in other plant species. Promoter analysis revealed enrichment of stress, hormone signaling, and development-related elements. Furthermore, expression profiling under heat stress revealed significant upregulation of several *HpHsfs*, suggesting their involvement in heat stress response. Subcellular localization assays of three representative proteins (*HpHsf3*, *HpHsf5*, and *HpHsf17*) in *Nicotiana benthamiana* epidermal cells confirmed their nuclear localization, supporting their function as transcription factors.

**Discussion:**

These findings provide new insights into the *HpHsf* gene family and lay a foundation for future functional studies on heat tolerance in alpine plants.

## 1 Introduction

Global climate change poses a significant threat to agricultural productivity by altering environmental conditions, highlighting the urgent need to elucidate plant responses to abiotic stressors, particularly heat stress (Sato et al., 2024). Elevated temperatures compromise plant health and performance, leading to stunted growth, reduced yields, and disrupted physiological functions. Alpine medicinal plants, adapted to cooler environments, are especially vulnerable to rising temperatures that impair their development and reproductive capacity (Zhu et al., 2024a). Understanding heat stress responses mechanisms in these high-altitude species is essential for formulating adaptive strategies to enhance their resilience and ensure sustainable cultivation in a warming climate.

Heat shock transcription factors (Hsfs) constitute a key family of transcription factors that serve as the central regulators of the heat shock response (Scharf et al., 2012). This response represents a highly conserved protective mechanism ubiquitously employed by organisms to mitigate various stresses, particularly thermal stress (Ohama et al., 2017; Wang and Xu, 2025). Structurally, Hsfs are characterized by several conserved functional domains, including a DNA-binding domain (DBD), an oligomerization domain (OD), a nuclear localization signal (NLS), a nuclear export signal (NES), a repressor domain (RD), and an aromatic, hydrophobic, and acidic (AHA) motif (Wang and Xu, 2025). Based on these structural features, Hsfs are generally classified into three major groups: A, B, and C. Class A Hsfs possess a C-terminal AHA motif that enables direct transcriptional activation of downstream target genes (Von Koskull-Döring et al., 2007). In contrast, Class B Hsfs contain a repressor domain with a highly conserved LFGV tetrapeptide motif (Ikeda and Ohme-Takagi, 2009). Class C Hsfs are generally shorter and structurally simpler than those in Classes A and B. Interestingly, the expansion of Class C Hsfs in monocots suggests potential functional specialization among members within this group (Andrási et al., 2020).

Plant Hsfs maintain a dynamic equilibrium, switching between active and inactive states in response to environmental conditions and adaptive mechanisms (Bakery et al., 2024). According to the activation cycle model, Hsfs are initially maintained in an inactive state within cytoplasmic complexes bound to HSP70 and HSP90. Under heat stress, misfolded proteins compete for and sequester HSPs, leading to Hsf release. Once liberated, Hsfs oligomerize, translocate into the nucleus, and activate the transcription of heat-responsive genes, including those encoding HSPs and additional Hsfs (Scharf et al., 2012; Bakery et al., 2024). In *Arabidopsis thaliana*, HsfA1 is a master regulator of high-temperature signaling, coordinating heat shock and warm-temperature responses. Specifically, HsfA1d links these pathways by stabilizing phytochrome-interacting factor 4 (PIF4), which promotes thermomorphogenesis, while simultaneously activating HsfA2 to reinforce thermotolerance (Raturi and Zinta, 2024; Li et al., 2024; Tan et al., 2023). Nevertheless, HsfBs are occasionally regarded as repressors of the heat shock response, capable of modulating the activity of class A members (Andrási et al., 2020; Wang and Xu, 2025). Loss-of-function mutations in *AtHsfB1* and *AtHsfB2b* confer enhanced heat tolerance in Arabidopsis, accompanied by elevated expression of *AtHsfA2* and *AtHsfA7a* under heat stress (Pick et al., 2012; Ikeda et al., 2011). In tomato, *SlHsfb1* acts as a co-activator of *SlHsfA1a*, promoting the accumulation of heat shock proteins and enhancing thermotolerance. In addition, *SlHsfb1* functions as a transcriptional repressor of other *SlHsfs*, including *SlHsfA1b* and *SlHsfA2*, thereby maintaining a balance between growth and stress responses under heat stress conditions (Fragkostefanakis et al., 2019). Furthermore, class C Hsfs contribute to the regulation of plant heat tolerance. For example, in lilies, high-temperature stress suppresses *LlHsfC2* homodimerization while enhancing its heterodimerization with HsfAs, thereby enabling sustained co-activation of heat tolerance mechanisms (Wu et al., 2024).

*Herpetospermum pedunculosum*, commonly known as ‘Bo-Leng-Gua’, is an annual species of Cucurbitaceae family, endemic to the Tibetan Plateau. It has garnered significant attention due to its high pharmacological value, particularly in traditional Tibetan medicine (Zhao et al., 2019). The seeds of *H. pedunculosum* are notably rich in lignan compounds (Zhu et al., 2024b), which exhibit diverse medicinal properties, including hepatoprotective, anti-cholestatic, and anti-inflammatory effects (Fang et al., 2007; Wei et al., 2021; Li et al., 2023a). These attributes underscore its importance within traditional Tibetan medicine. Ecologically, *H. pedunculosum* is well adapted to the extreme conditions of high-altitude habitats, typically occurring between 2,300 and 3,500 m, where it withstands such as strong winds, low temperatures, drought, and hypoxic stress (Zhao et al., 2019; Chen et al., 2025). However, with ongoing climate change and increasing global temperatures, its long-term survival and medicinal utility may be at risk. Thus, elucidating the mechanisms underlying its thermotolerance is crucial not only for understanding its ecological resilience but also for informing future conservation, cultivation, and sustainable utilization strategies.

Hsfs play a central role in plant responses to high-temperature stress by regulating the expression of heat shock proteins and other protective genes (Scharf et al., 2012; Kan et al., 2023). Investigating the *Hsf* gene family in *H. pedunculosum* offers valuable insights into its thermotolerance mechanisms and adaptive evolution, while providing a molecular basis for developing stress-resilient cultivars. In this study, we comprehensively characterize the *HpHsf* gene family in *H. pedunculosum* and analyze their expression patterns under heat stress conditions, thereby advancing the broader understanding of thermotolerance mechanisms in plants.

## 2 Materials and methods

### 2.1 Identification of *Hsf* genes from *H. pedunculosum*

The complete genome sequence of *H. pedunculosum* was obtained from Figshare (https://doi.org/10.6084/m9.figshare.21626153.v2) (Yang et al., 2023). Hidden Markov Model (HMM)-based screening was employed to identify *Hsf* genes. The Hsf domain profile (PF00447) was retrieved from the Pfam database, and putative Hsfs were identified using HMMER v3.0 with a significance threshold of E-value < 1 × 10^-5^ (Potter et al., 2018; Mistry et al., 2020). Candidate Hsf protein sequences were subsequently validated using the NCBI Batch CD-Search tool (https://www.ncbi.nlm.nih.gov/Structure/bwrpsb/bwrpsb.cgi).

### 2.2 Sequence analyses of *HpHsfs*

Protein physicochemical properties and gene structures were analyzed using TBtools (Chen et al., 2023). Subcellular localization was predicted using WOLF PSORT (https://www.genscript.com/wolf-psort.html). Conserved motifs were identified with MEME Suite v5.5.8 (https://meme-suite.org/meme/) (Bailey et al., 2009), while conserved domains within protein sequences were predicted using the NCBI Batch Web CD-Search tool (https://www.ncbi.nlm.nih.gov/Structure/bwrpsb/bwrpsb.cgi). Key amino acid motifs were identified from a ClustalW-based multiple sequence alignment of all HpHsf proteins, with reference to the established characteristics of Hsf signature motifs (Scharf et al., 2012).

### 2.3 Phylogenetic analysis of the *HpHsf* gene family

For phylogenetic analysis and subfamily classification, full-length Hsf amino acid sequences from *Cucumis sativus*, *Arabidopsis thaliana*, and *Oryza sativa* were retrieved using a consistent approach. Multiple sequence alignment was performed with ClustalW, and phylogenetic trees were constructed in MEGA 12 using the neighbor-joining method with 1000 bootstrap replicates and default settings (Substitution type: Amino acid; Model/Method: Jones-Taylor-Thornton (JTT) model; Rates among Sites: Uniform Rates; Gaps/Missing Data Treatment: Patial deletion, 50%) (Tamura et al., 2021). The resulting subfamily clustering was consistent with the previously established classification of the *C. sativus Hsf* family (Chen et al., 2021). Phylogenetic trees were visualized using the ChiPlot online tool (https://www.chiplot.online/tvbot.html) (Xie et al., 2023).

### 2.4 Analysis of *cis*-regulatory elements of *HpHsfs*

Using the Gtf/Gff3 Sequences Extractor in TBtools, 2,000 bp promoter regions upstream of the ATG start codon of all *H. pedunculosum Hsf* genes were extracted from the genome. Cis-acting regulatory elements within these sequences were identified using the PlantCARE web server (http://bioinformatics.psb.ugent.be/webtools/plantcare/html/) (Lescot et al., 2002), and the results were visualized with TBtools’ Simple BioSequence Viewer.

### 2.5 Chromosome distribution and gene collinearity analysis of *HpHsf* gene family

Based on the *H. pedunculosum* genome annotation, we retrieved chromosomal localization information for *HpHsf* genes, generated a chromosomal distribution map with TBtools, and designated gene names according to their positional order along the chromosomes. Intraspecific collinearity analysis of *Hsf* genes in *H. pedunculosum* was performed using MCScanX integrated into TBtools, and the results were visualized with the Advanced Circos module. Genome data for *A. thaliana* were obtained from The Arabidopsis Information Resource (TAIR; http://www.arabidopsis.org) (Lamesch et al., 2011), while those for *C. sativus* were retrieved from the Cucurbit Genomics Database (http://cucurbitgenomics.org/) (Li et al., 2019). Interspecific collinearity analysis between *H. pedunculosum* and the two reference species was also conducted using MCScanX in TBtools.

### 2.6 Plant materials and heat stress

Seeds of *H. pedunculosum* were sourced from the cultivation base of Tibet Rhodiola Pharmaceutical Holding Company in Lhasa (29°79′ N, 94°09′ E, Nyingchi, Xizang, China). These seeds were morphologically authenticated by Prof. Qi Zhao from Chengdu University and subsequently stored at the Engineering Research Center of Sichuan-Xizang Traditional Medicinal Plants, Chengdu, China. Seedlings were hydroponically grown in Hoagland solution under controlled growth chamber conditions (24°C day/22°C night, 16-h photoperiod) for 40 days. Uniform seedlings were subjected to heat stress at 42°C (Xu et al., 2006; Chen et al., 2021), and leaf samples were collected at 0 (control), 1, 3, 6, and 12 hours post-treatment. All samples were immediately frozen in liquid nitrogen and stored at -80°C until RNA extraction.

### 2.7 RNA extraction, qRT-PCR, and RT-PCR analysis

Leaf samples of *H. pedunculosum* were homogenized using an ALLSHENG Bioprep-24R homogenizer (Hangzhou, China). Subsequently, total RNA was extracted from the homogenized tissue using a plant total RNA extraction kit (Foregene Biotechnology, Chengdu, China) in accordance with the manufacturer’s instructions. cDNA was synthesized using the HiFiScript gDNA Removal RT MasterMix (Cwbio, Jiangsu, China). The expression levels of *HpHsfs* were quantified using quantitative real-time PCR (qRT-PCR) using the Cwbio SYBR Master Mix kit (Cwbio, Jiangsu, China) on a Bio-Rad CFX96 Real-time PCR system, with the *H. pedunculosum Actin* gene serving as the internal control. The primer sequences are provided in **Supplementary Table 1**. Statistical analysis was performed using one-way analysis of variance (ANOVA) in SPSS 26.0. Data obtained from three or four independent technical replicates are presented as the mean ± standard deviation (SD). The results were verified in two independent biological experiments. Significant differences (*p* < 0.05) were determined using Tukey’s multiple comparisons test and are indicated by distinct superscript letters. The raw data are provided in **Supplementary Table 3**.

For RT-PCR, first-strand cDNA synthesis was performed with the BioRT All-in-One RT Master Mix for qPCR (Bioer Technology, Hangzhou, China). We amplified the PCR products of *HpHsfs* splice variants with Green Taq Mix (Vazyme, Nanjing, China) and resolved them on a 2% agarose gel. The corresponding primer sequences can be found in **Supplementary Table 1**. All assays were repeated at least twice and yielded concordant results.

### 2.8 Subcellular localization analysis

The open reading frames (ORFs) of *HpHsf3*, *HpHsf5*, and *HpHsf17* without stop codons were amplified using primers containing *Bam*H I and *Kpn* I sites (**Supplementary Table 1**) and subcloned in-frame into the pCAMBIA1300-35S-YFP vector. The RFP-NLS_SV40_ fusion protein was utilized to label the nucleus (Huang et al., 2014), while the empty vector served as a negative control. All constructs were individually transformed into the *Agrobacterium tumefaciens* strain GV3101. Following the protocol described by (Li et al., 2023b), *Agrobacterium* strains harboring the plasmids were infiltrated into *Nicotiana benthamiana* leaves, and fluorescence signals were observed under a laser confocal microscope two days post-transient expression (Nikon Ti2-E AXR+NSPARC, Tokyo, Japan).

## 3 Results

### 3.1 Identification and characterization of the physical properties of *Hsf* gene family members in *H. pedunculosum*

**Figure 1:**
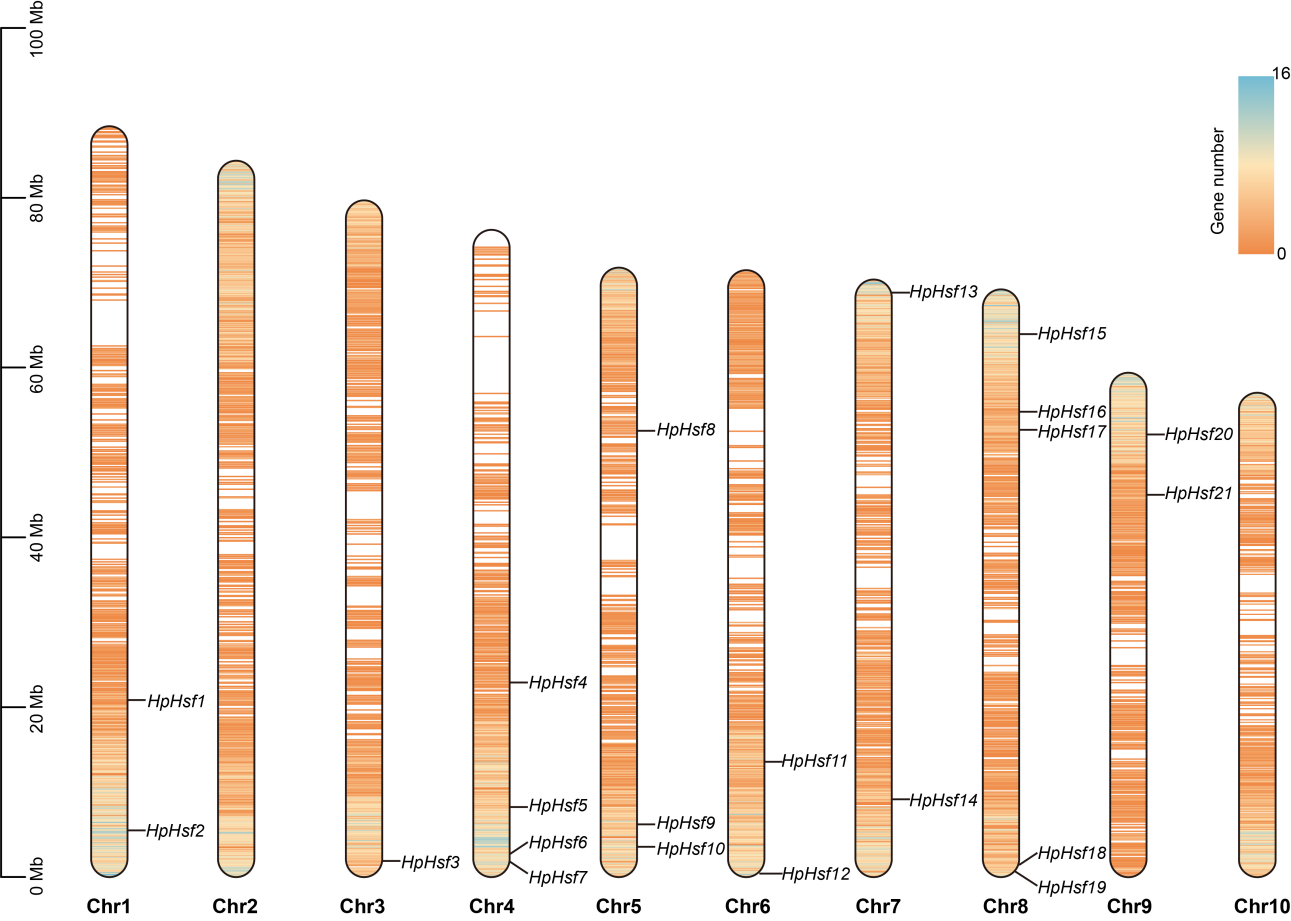
Chromosomal distribution of *HpHsfs* in *H. pedunculosum*. The ruler on the left represents the length of the chromosome. Regions of higher gene density are depicted in blue, contrasting with lower-density regions rendered in orange.

A total of 21 *HpHsf* genes were identified in the *H. pedunculosum* genome and designated *HpHsf1* through *HpHsf21* according to their chromosomal starting positions (**Table 1**, **Figure 1**). These genes were unevenly distributed across eight of the 10 chromosomes. Analysis of their physicochemical properties revealed predicted ORF lengths ranging from 549 bp (*HpHsf6*) to 1566 bp (*HpHsf11*), corresponding to protein lengths of 182–521 amino acids. The molecular weights of the encoded proteins varied from 21.43 kDa to 56.99 kDa, while their theoretical isoelectric points (pI) ranged from 4.68 (HpHsf1) to 9.23 (HpHsf8). All HpHsf proteins exhibited negative grand average of hydropathicity (GRAVY) values, suggesting hydrophilic characteristics. Subcellular localization analysis further predicted that all HpHsf proteins are localized in the nucleus.

**Table 1:** The detailed information of HpHsf members.

Gene name	Gene ID	Chr	CDS (bp)	Exon	AA	MW (kDa)	pI	GRAVY	Class	Key motifs	Subcellular localization
*HpHsf1*	Hsped.01g06140.1	1	1239	2	412	47.80	4.68	-0.727	A	DBD, OD, AHA, NES	Nucleus
*HpHsf2*	Hsped.01g17750.1	1	1038	2	345	39.17	7.26	-0.706	B	DBD, OD, RD, NLS, NES	Nucleus
*HpHsf3*	Hsped.03g20340.1	3	1128	2	375	43.20	5.19	-0.72	A	DBD, OD, NLS, AHA, NES	Nucleus
*HpHsf4*	Hsped.04g04010.1	4	702	2	233	26.88	6.04	-1.002	B	DBD, OD, RD	Nucleus
*HpHsf5*	Hsped.04g12450.1	4	894	2	297	33.29	5.17	-0.975	B	DBD, OD, RD, NLS	Nucleus
*HpHsf6*	Hsped.04g18480.1	4	549	2	182	21.43	6.46	-0.724	B	DBD, OD	Nucleus
*HpHsf7*	Hsped.04g19380.1	4	1203	2	400	46.30	5.69	-0.94	A	DBD, OD, NLS, AHA, NES	Nucleus
*HpHsf8*	Hsped.05g08010.1	5	762	2	253	29.17	9.23	-0.847	B	DBD, OD, RD, NLS	Nucleus
*HpHsf9*	Hsped.05g15860.1	5	1038	3	345	40.25	5.11	-0.99	A	DBD, OD, NLS, AHA, NES	Nucleus
*HpHsf10*	Hsped.05g17840.1	5	1404	2	467	52.61	6.61	-0.757	A	DBD, OD, NLS, AHA, NES	Nucleus
*HpHsf11*	Hsped.06g10010.1	6	1566	3	521	56.99	5.08	-0.494	A	DBD, OD, NLS, AHA, NES	Nucleus
*HpHsf12*	Hsped.06g19760.1	6	1047	4	348	40.03	5.78	-0.779	A	DBD, OD, NLS, AHA, NES	Nucleus
*HpHsf13*	Hsped.07g01730.1	7	834	2	277	31.55	6.92	-0.635	B	DBD, OD, NLS	Nucleus
*HpHsf14*	Hsped.07g18350.1	7	1233	2	410	46.80	5.4	-0.628	A	DBD, OD, NLS, AHA, NES	Nucleus
*HpHsf15*	Hsped.08g06000.1	8	924	2	307	35.36	6.22	-0.776	A	DBD, OD, NLS, AHA, NES	Nucleus
*HpHsf16*	Hsped.08g12920.1	8	1110	2	369	41.35	7.78	-0.515	B	DBD, OD, RD, NLS, NES	Nucleus
*HpHsf17*	Hsped.08g13990.1	8	1080	5	359	41.12	4.95	-0.565	A	DBD, OD, NLS, AHA, NES	Nucleus
*HpHsf18*	Hsped.08g25890.1	8	987	2	328	37.17	7.74	-0.563	C	DBD, OD	Nucleus
*HpHsf19*	Hsped.08g26700.1	8	1212	2	403	45.75	6.05	-0.6	A	DBD, OD, NLS, AHA	Nucleus
*HpHsf20*	Hsped.09g07540.1	9	870	2	289	32.78	5.31	-0.599	C	DBD, OD, NLS	Nucleus
*HpHsf21*	Hsped.09g11810.1	9	732	2	243	28.20	7.22	-0.638	B	DBD, OD, RD, NLS	Nucleus

Chr, Chromosome; CDS, length of coding sequence; AA, number of amino acids; MW, molecular weight; pI, theoretical isoelectric point; GRAVY, grand average of hydropathicity; DBD, DNA-binding domain, OD, oligomerization domain; NLS, nuclear localization signal; AHA, aromatic, hydrophobic, and acidic; NES, nuclear export signal; RD, repressor domain.

### 3.2 Chromosomal localization and phylogenetic analysis of the *HpHsf* gene family

**Figure 2:**
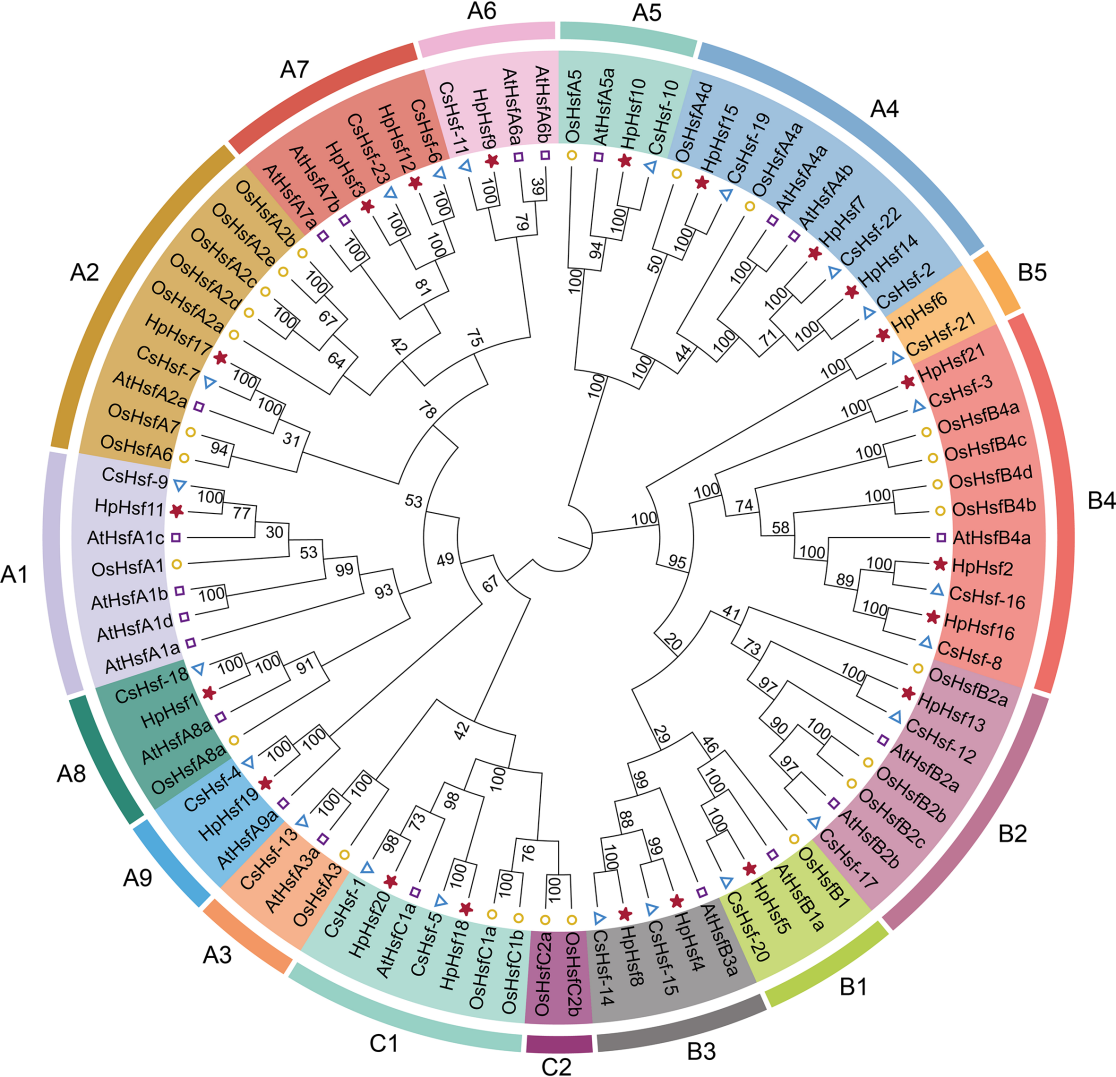
Phylogenetic analysis of *Hsf* gene family members in *H. pedunculosum* (Hp), *A.thaliana* (At), *O. sativa* (Os), and *Cucumis sativus* (Cs). Distinct colors represent different *Hsf* subfamilies.

Chromosomal distribution analysis revealed that the 21 *HpHsf* genes are unevenly distributed across chromosomes 1 and 3 to 9. Among these, chromosome 8 exhibited the highest number, with five *HpHsf* genes, whereas chromosome 3 harbored only a single gene (**Figure 1**). We performed a phylogenetic analysis of the *Hsf* gene family in *H. pedunculosum* using amino acid sequences from 21 HpHsf proteins, 21 AtHsf proteins (*A. thaliana*), 25 OsHsf proteins (*O. sativa*), and 23 CsHsf proteins (*C. sativus*) (**Figure 2**; **Supplementary Table 2**). Based on this analysis, Hsf proteins were classified into three subfamilies: HsfA, HsfB, and HsfC (Shamshad et al., 2023; Guo et al., 2008; Chen et al., 2021). Class A was further divided into nine subtypes (A1-A9) comprising 11 members, while Class B was segregated into five subtypes (B1-B5) containing eight members. Class C was separated into two subtypes (C1-C2), of which only C1 was represented, with a single member. The phylogenetic tree showed that multiple HpHsfs clustered closely with CsHsfs. In addition, members of the AtHsf, HpHsf, and CsHsf subfamilies were absent from subtype C2.

### 3.3 Gene duplication and synteny analysis of *HpHsf* genes

**Figure 3:**
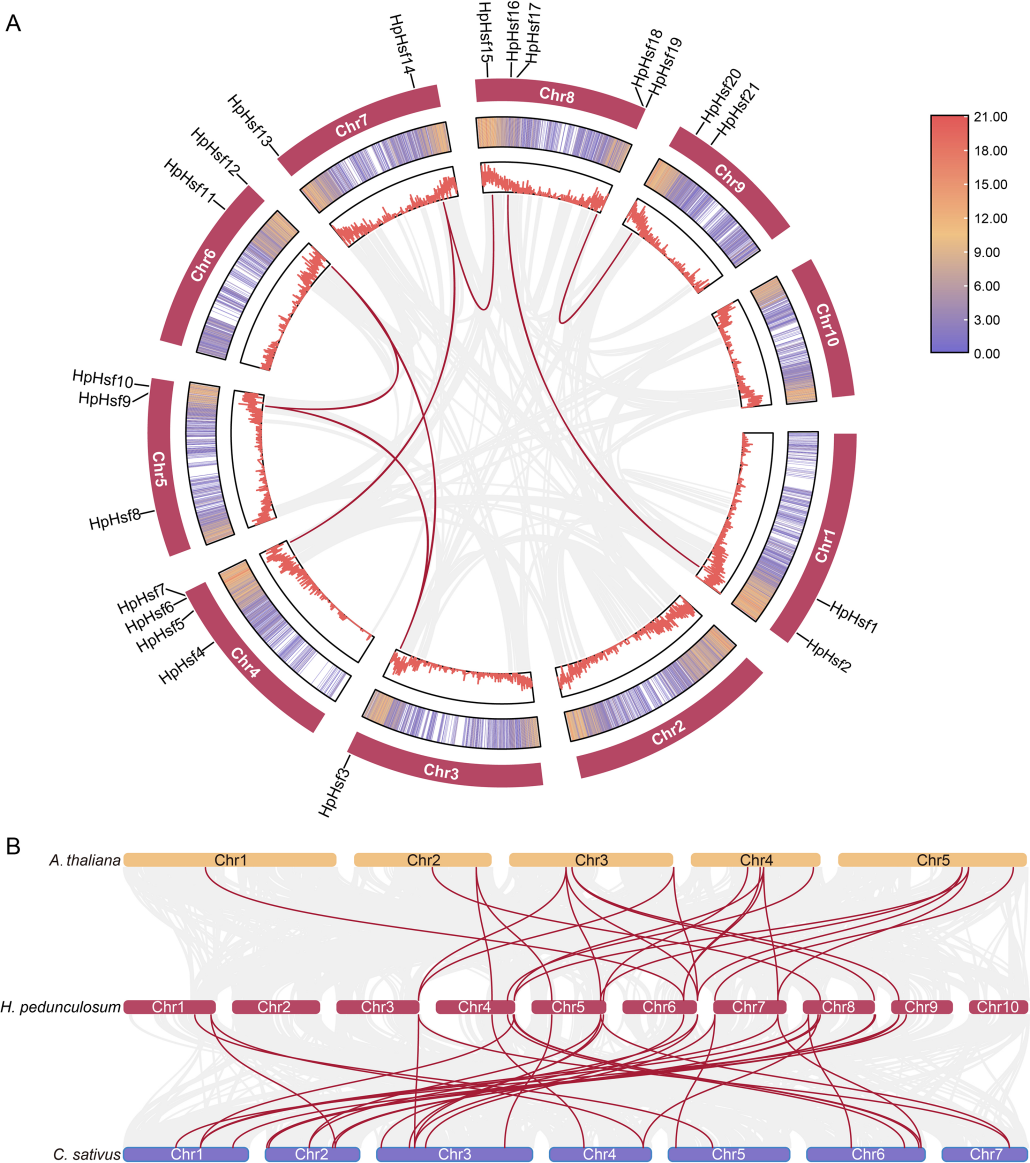
Collinearity analysis of *H pedunculosum*. **(A)** Collinearity among *HpHsf* members in *H pedunculosum*. Red lines highlight duplicated *HpHsf* gene pairs, whereas the gray line represents all duplicate gene pairs. **(B)** Comparative synteny analysis of *Hsf* genes between *H pedunculosum*, *A thaliana*, and*C sativus*. Background gray lines indicate collinear blocks between *H pedunculosum* and other plant genomes, with *Hsf*-related gene pairs highlighted in red.

Gene duplication is a key evolutionary mechanism in plants, contributing to genetic diversity and driving the development of novel traits such as stress resistance and floral complexity (Panchy et al., 2016). Intraspecific collinearity analysis identified eight segmental duplication pairs among the *HpHsfs* genes, including *HpHsf2*/*HpHsf16*, *HpHsf3*/*HpHsf9*, *HpHsf3*/*HpHsf12*, *HpHsf7*/*HpHsf14*, *HpHsf9*/*HpHsf12*, *HpHsf14*/*HpHsf15*, and *HpHsf18*/*HpHsf20* (**Figure 3A**). Notably, *HpHsf3*, *HpHsf9*, and *HpHsf12* exhibited linear correlations, while *HpHsf14* correlated with *HpHsf7* and *HpHsf15*. These findings suggest that gene duplication events have contributed to the expansion of the *HpHsf* family and likely played a significant role in its evolutionary diversification.

To further investigate the evolutionary patterns of the *HpHsf* gene family, synteny analysis was conducted between *H. pedunculosum* and two representative species, *A. thaliana* and *C. sativus* (**Figure 3B**). The analysis identified 22 collinear gene pairs with *A. thaliana* and 32 with *C. sativus*, suggesting a closer phylogenetic relationship between *H. pedunculosum* and cucumber than with Arabidopsis. In addition, 15 *HpHsf* genes showed collinearity with both *A. thaliana* and *C. sativus*, indicating that these genes represent conserved orthologs inherited from a common ancestor. Notably, *HpHsf9* and *HpHsf12* were associated with two to three collinear gene pairs in both species, implying potential gene duplication events and a shared evolutionary origin for these loci.

### 3.4 Analysis of the *HpHsf* gene structures, conserved motifs, and conserved domains

**Figure 4:**
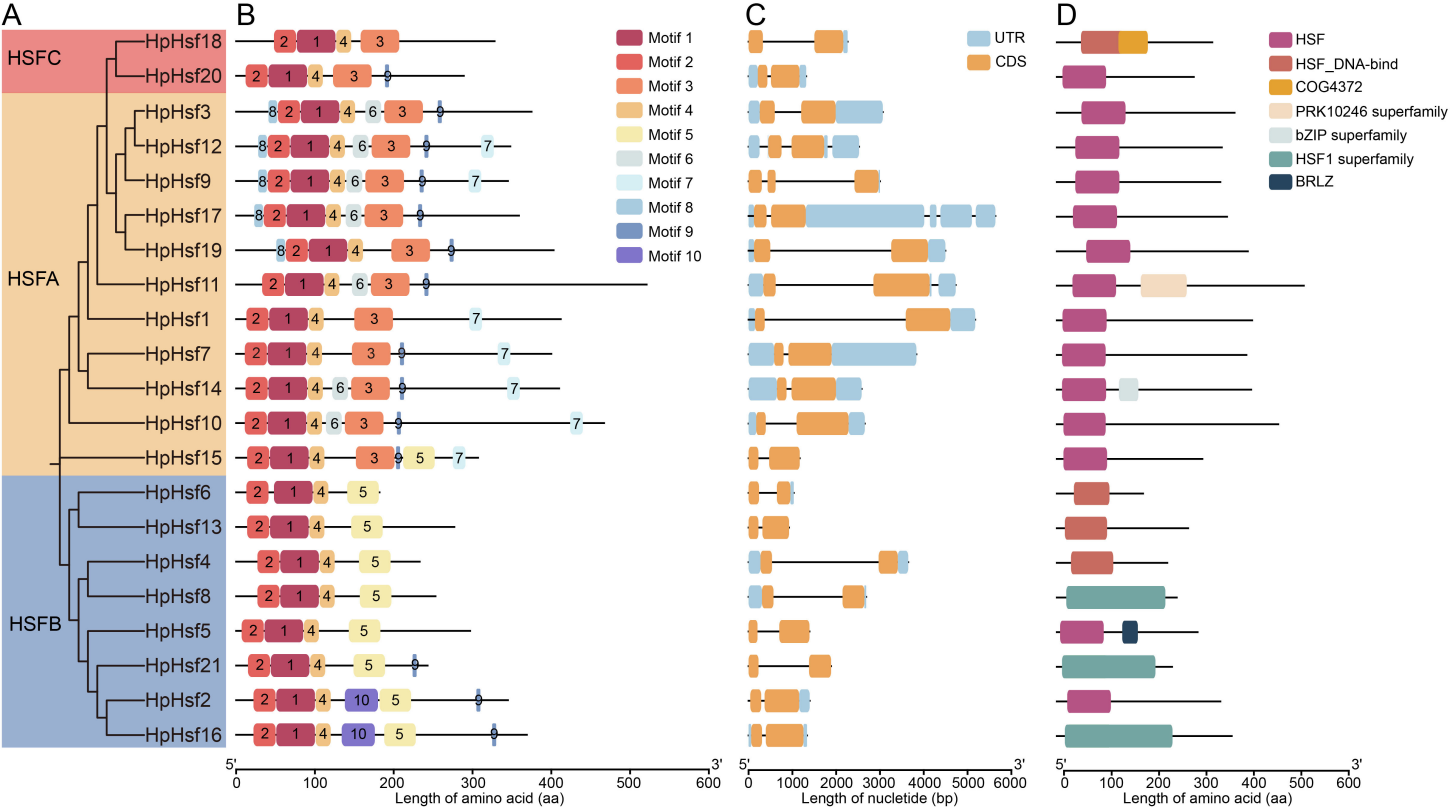
Phylogenetic relationship, motif distribution, gene structure and conserved domain of HpHsf proteins. **(A)** Phylogenetic relationships of HpHsf proteins, with different colors representing distinct subgroups. **(B)** Motif composition of HpHsf members, with each of the 10 motifs depicted by uniquely colored boxes. **(C)** Gene structures of *HpHsf* gene family. **(D)** Conserved domain structures of HpHsf proteins.

We conducted a comprehensive characterization of the *HpHsf* family by analyzing phylogenetic relationships, motif patterns, gene structures, and conserved domains (**Table 1**; **Figure 4**). A total of ten conserved motifs were identified, of which motifs 2, 1, and 4 were present in all members (**Figure 4B**). Motifs 6, 7, and 8 were exclusively detected in Class A Hsfs, whereas Motifs 5 and 10 were specific to Class B, with the exception of HpHsf15. In contrast, Class B members lacked motif 3, which was consistently observed in both Class A and Class C. Further characterization of canonical Hsf motifs revealed distinct architectural features among the three classes (**Table 1**). Class A proteins were defined by a canonical suite of motifs (DBD, OD, NLS, AHA, and NES), with the exceptions of HpHsf1 and HpHsf19, which lacked the NLS and NES, respectively. The RD motif was ubiquitous in Class B members, except for HpHsf6 and HpHsf13. Class C members exhibited the simplest architecture, typically possessing only the DBD and OD, occasionally accompanied by an NLS. Taken together, these results demonstrate that the Hsf family in *H. pedunculosum* conforms to the fundamental structural framework typical of plant HSF proteins. Moreover, the distinct motif signatures observed among the three subfamilies reflect clear structural and functional divergence, supporting their phylogenetic classification.

Gene structure analysis revealed that *HpHsf17* was the only member containing three exons, whereas the other *HpHsf* genes consisted of two exons (**Figure 4C**). Furthermore, conserved domain analysis showed that all Class A members contain the HSF domain, while Class B members display a broader array of domains, including HSF, HSF_DNA-bind, HSF1 superfamily, and BRLZ. This pattern implies that Class A domains have remained more conserved throughout evolution, whereas Class B members exhibit greater domain diversification (**Figure 4D**).

### 3.5 Analysis of cis−acting elements in the promoter of *HpHsf* genes

**Figure 5:**
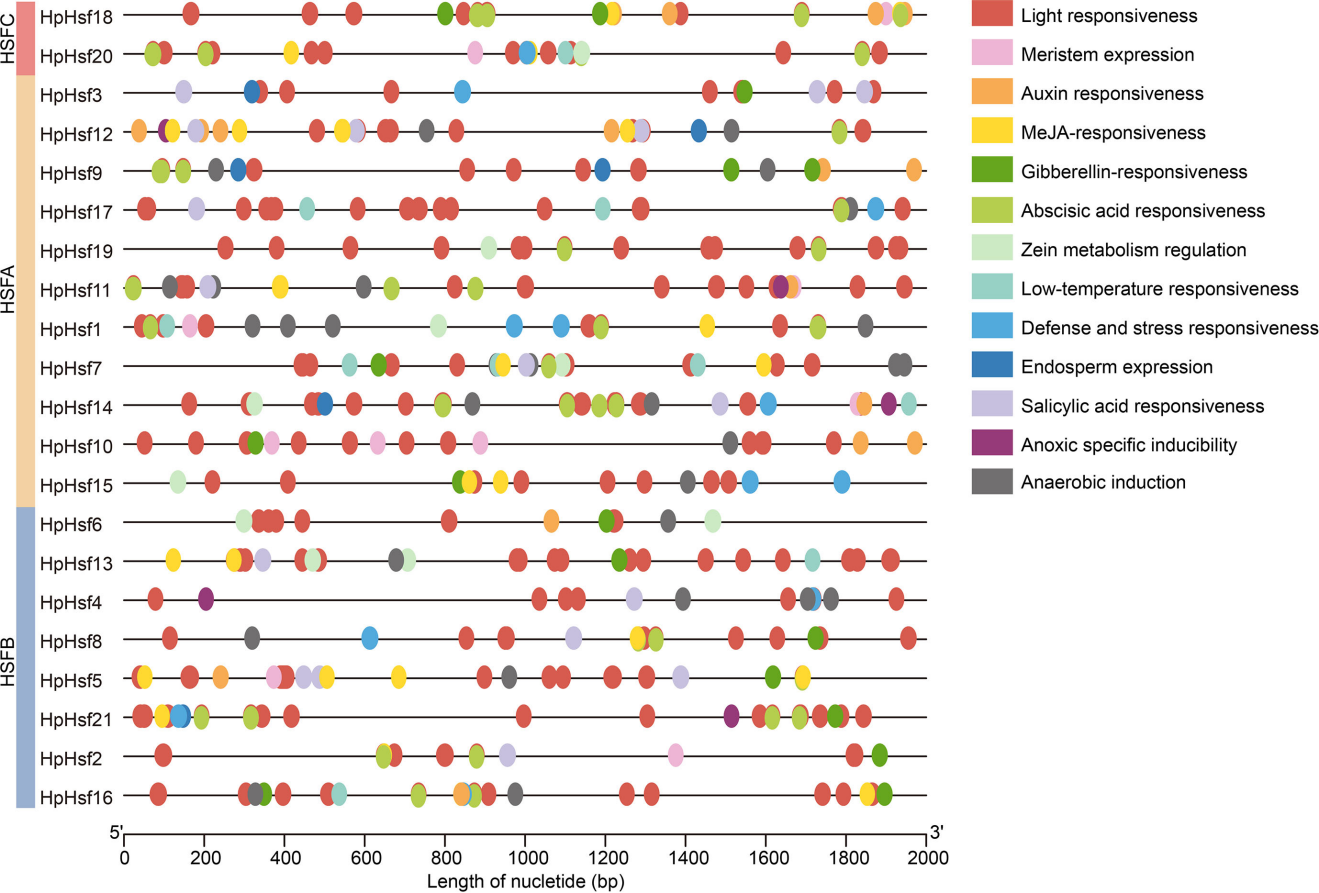
Analysis of cis-acting elements in the *HpHsf* promoters. Different colored squares represent distinct cis-acting elements within the *HpHsf* promoter regions.

To investigate the potential regulatory mechanisms and functional roles of *HpHsf* genes, the 2,000 bp upstream sequences from the translation start site were analyzed using the PlantCARE database to identify putative cis-acting regulatory elements. The promoter analysis revealed that the *HpHsf* gene family contains various cis-acting elements associated with stress response (low-temperature responsiveness, defense and stress responsiveness, anoxic specific inducibility, and anaerobic induction), hormonal signaling pathways (auxin responsiveness, MeJA-responsiveness, gibberellin-responsiveness, abscisic acid responsiveness, zein metabolism regulation, and salicylic acid responsiveness), and developmental processes (light responsiveness, meristem expression, and endosperm expression) (**Figure 5**). Notably, all 21 *HpHsf* gene promoters contained multiple light-responsive elements, while the distribution of stress- and development-related elements varied among members. These findings suggest that the *HpHsf* gene family plays a crucial role in light-response regulation and possesses diverse regulatory potential in other biological processes.

### 3.6 Expression patterns of *HpHsfs* under heat stress and subcellular localization of three HpHsfs

**Figure 6:**
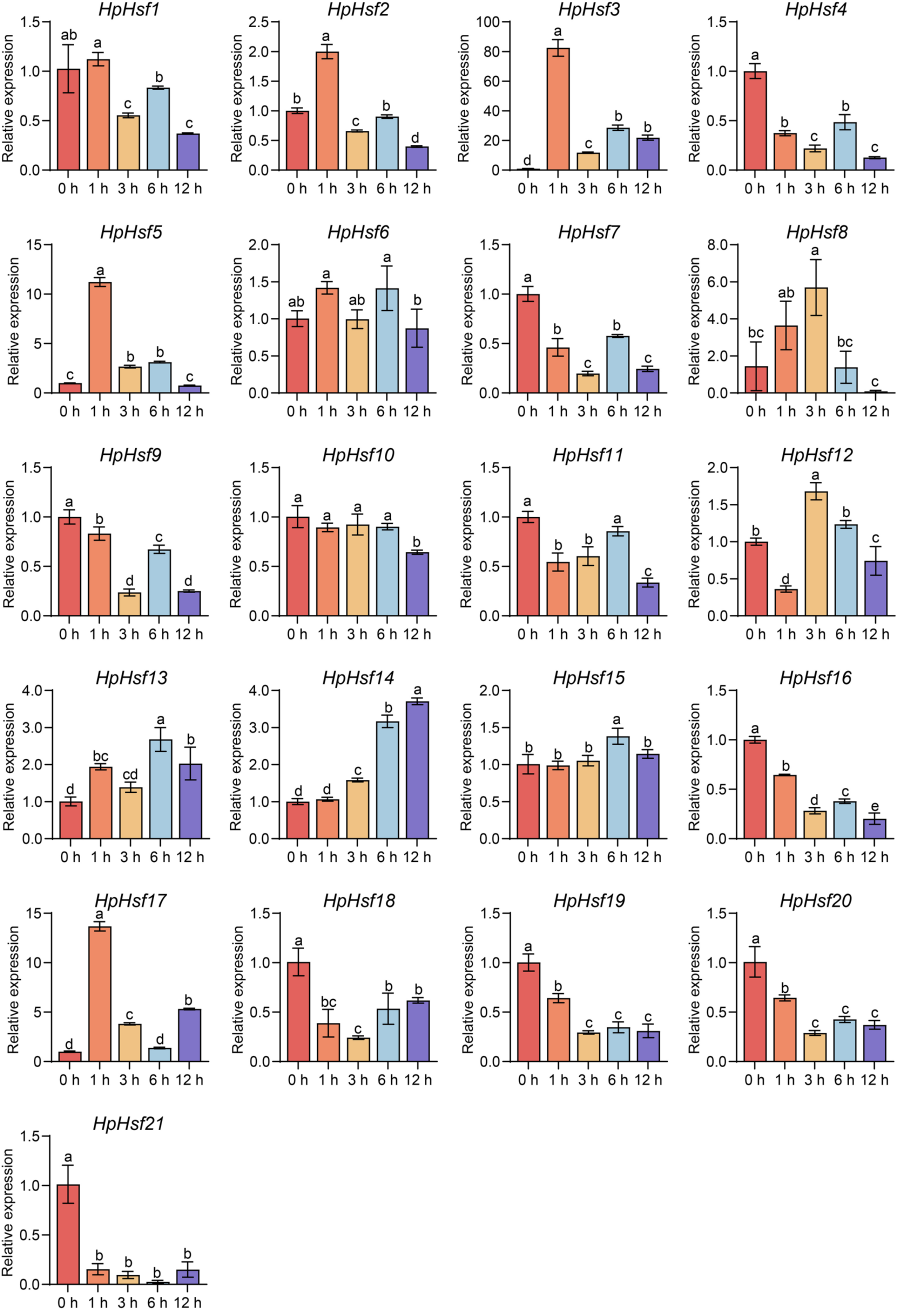
The relative expression levels of *HpHsfs* in response to heat stress. Transcript levels of *HpHsfs* were analyzed by qRT-PCR in *H. pedunculosum* leaves subjected to heat stress (42 °C) at different time points (0 h, 1 h, 3 h, 6 h, and 12 h). Samples collected at 0 h served as the normalization control. Data are mean ± SD from three or four independent experiments. Significance was determined via one-way ANOVA with Tukey’s multiple comparisons test. Different letters indicate significant differences at *p* < 0.05. All experiments were performed with two biological replicates.

**Figure 7:**
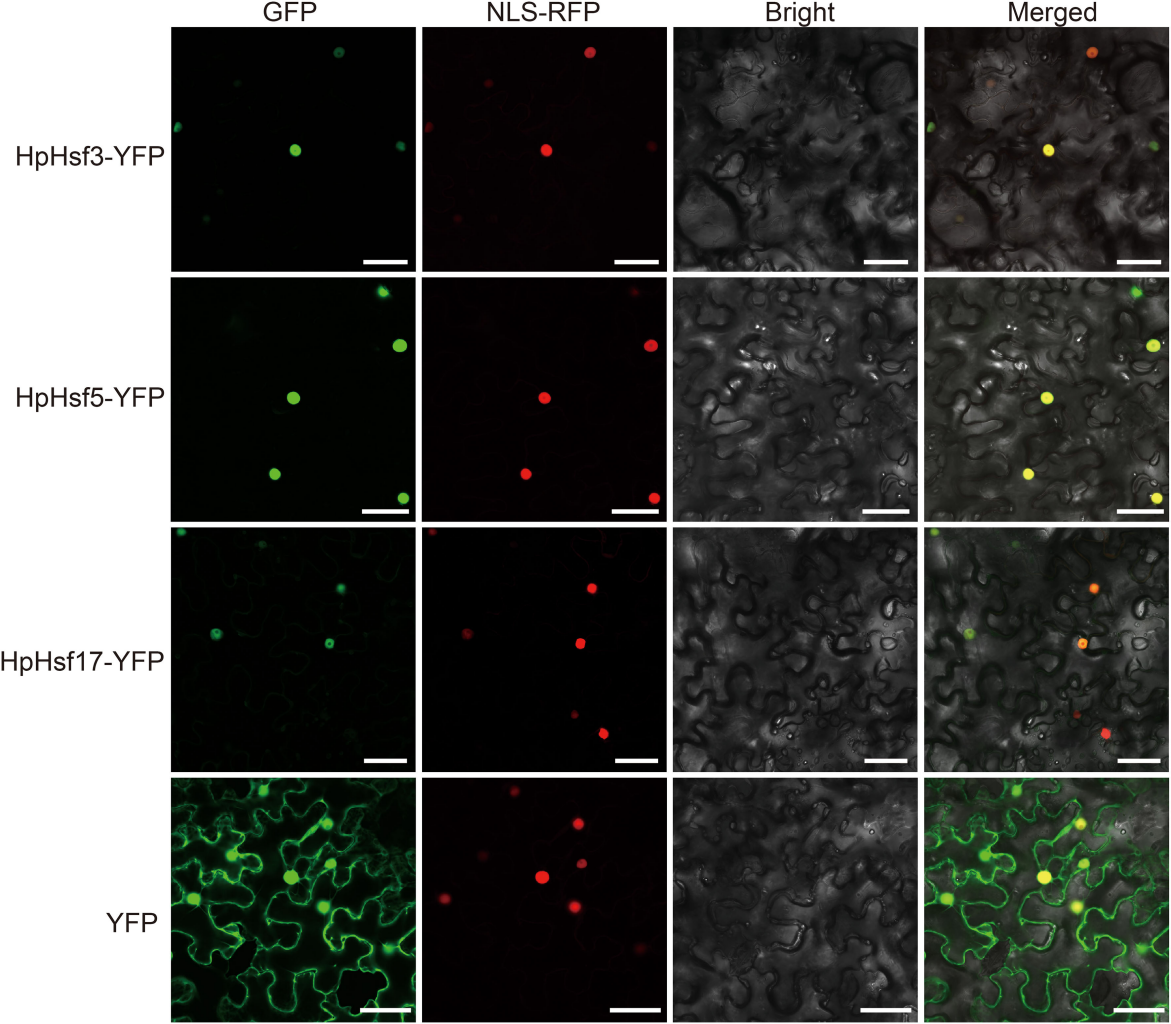
Subcellular localization of HpHsf3, HpHsf5, and HpHsf17 in *N. benthamiana* leaves. The transient expression of p35S::HpHsf3-YFP, p35S::HpHsf5-YFP, and p35S::HpHsf17-YFP, was observed in *N. benthamiana* leaves, using the YFP empty vector as a control. The RFP-NLS_SV40_ fusion protein served as a nuclear marker. Scale bars, 50 μm.

Extensive evidence indicates that *Hsfs* play key roles in plant responses to high temperatures (Kan et al., 2023; Ding et al., 2020). Here, we characterized the expression profiles of all 21 *HpHsfs* under heat stress at different time points (0, 1, 3, 6, and 12 h) using qRT-PCR (**Figure 6**). *HpHsf2*, *HpHsf3*, *HpHsf5*, and *HpHsf17* exhibited transient upregulation, with transcript levels peaking at 1 h and declining by 3 h, indicating a rapid transcriptional response. Among these, *HpHsf3* and *HpHsf17* belong to the Class A subfamily, while *HpHsf2* and *HpHsf5* are categorized into the Class B subfamily. Notably, the expression of *HpHsf3* and *HpHsf17* showed signs of reactivation at 12 h after heat stress. These results imply that these genes may function as critical regulators in the heat stress response of *H. pedunculosum*. To further validate their functional roles, we examined the subcellular localization of *HpHsf3, HpHsf5*, and *HpHsf17* (**Figure 7**). The YFP-tagged HpHsf proteins were exclusively localized in the nucleus, colocalizing with a nuclear marker. In contrast, the free YFP control was distributed throughout the entire cell. These results confirm the nuclear localization of these proteins, which is consistent with the predicted localization patterns.

To investigate alternative splicing of *HpHsf* genes under heat stress, we analyzed their transcript variants by RT-PCR (**Supplementary Figure 1**). Multiple amplicons were detected for *HpHsf1*, *HpHsf3*, *HpHsf5*, *HpHsf6*, *HpHsf9*, *HpHsf10*, and *HpHsf19*, suggesting that these genes potentially undergo alternative splicing in *H. pedunculosum*. However, no heat stress-induced splicing variants were observed. This indicates that alternative splicing is unlikely to be a primary regulatory mechanism for these *HpHsf* genes in response to heat stress.

## 4 Discussion

Given the pivotal role of the *Hsf* gene family in mediating plant thermotolerance, its composition and functions have been extensively characterized in several model plants and major crops, including Arabidopsis (Guo et al., 2008), rice (Shamshad et al., 2023), wheat (Zhou et al., 2019), and maize (Lin et al., 2011). Systematic identification and analysis of *Hsf* genes have been reported within the cucurbit family in species such as cucumber (Chen et al., 2021) and pumpkin (Shen and Yuan, 2020). However, in the medicinal cucurbit *H. pedunculosum*, the *Hsf* gene family remains largely unexplored, and its genomic organization and expression patterns under heat stress are still unclear.

In this study, 21 members of the *HpHsf* family were identified in *H. pedunculosum* and designated *HpHsf1* to *HpHsf21* according to their chromosomal locations (**Table 1**). Compared with cucumber (23 genes) and pumpkin (36 genes), *H. pedunculosum* harbors a smaller number of *Hsf* genes. This difference is likely due to variations in whole-genome duplication events, followed by lineage-specific gene loss or retention (Jiang et al., 2013). We further characterized the physicochemical properties of HpHsf (**Table 1**). The isoelectric point (pI) of HpHsf proteins ranged from 4.68 to 9.23, a distribution comparable to that reported for CsHsf proteins from cucumber (Chen et al., 2021) and CmHsf proteins from pumpkin (Shen and Yuan, 2020). This conserved pI range suggests that the charge variability may be a common feature of the Hsf proteins across cucurbitaceous plants. Furthermore, all HpHsf proteins exhibited negative GRAVY values, suggesting their hydrophilic nature and likely solubility in the aqueous cellular environment. This observation aligns with the water-soluble properties of Hsf proteins reported in other plant species (Liao et al., 2023; Li et al., 2025). All HpHsfs proteins are predicted to localize in the nucleus, consistent with the canonical subcellular distribution of transcription factors.

Phylogenetic analyses have shown that the plant *Hsf* gene family is evolutionarily conserved and can be classified into three major groups: A, B, and C (Guo et al., 2016; Scharf et al., 2012). In cucumber, the *CsHsf* family contains 23 genes, categorized into classes A (12), B (9), and C (2), with each class comprising several phylogenetically distinct subclades (Chen et al., 2021). In pumpkin, the classification is more intricate: 36 *CmHsf* genes are divided into three subfamilies (I, II, and III) according to phylogenetic analysis, which correspond to the conventional groups A, B, and C based on conserved structural characteristics. Specifically, subfamily II aligns with group A, subfamily III corresponds to group B, and subfamily I includes genes from groups A and C (Shen and Yuan, 2020). Herein, phylogenetic analysis of *Hsf* members from *H. pedunculosum*, *A. thaliana*, *O. sativa*, and *C. sativus* revealed a conserved clustering pattern, with genes grouped into three major classes (A, B, and C) and 15 subgroups (A1-A9, B1-B5, and C1) (**Figure 2**). This classification is consistent with previous reports in *H. pedunculosum* and cucumber (Chen et al., 2021). The results suggest that orthologous genes with similar motif composition may exhibit functional redundancy, while paralogous genes within the same lineage may retain overlapping functions. Notably, no Class C2 subfamily members were identified in *H. pedunculosum*. This indicates that C2-type Hsfs are absent in this dicot species, a finding consistent with phylogenetic patterns where C2-type Hsfs are typically restricted to monocots (Andrási et al., 2020). Collectively, these results reinforce the link between the functional diversification of *Hsf* subfamilies and the evolutionary history of plant taxa, offering valuable insights for future research on the molecular mechanisms underlying stress resistance in *H. pedunculosum*.

Eukaryotic genomes vary in the extent to which genes are retained on homologous chromosomes (synteny) and in the preservation of their linear order (collinearity) over evolutionary timescales (Tang et al., 2008). Gene duplication promotes genetic diversification within species, while collinearity analyses provide insights into the evolutionary trajectories and functional divergence of genes. This study identified eight segmentally duplicated pairs among the *HpHsf* genes in *H. pedunculosum*, encompassing 10 genes distributed across eight chromosomes (**Figure 3A**). This number is slightly lower than that reported in *Cucurbita moschata* (nine pairs) and *C. sativus* (13 pairs) (Shen and Yuan, 2020; Chen et al., 2021). These results suggest that segmental duplication has likely contributed significantly to the expansion of the *Hsf* gene family in *H. pedunculosum* and potentially across the Cucurbitaceae family. Moreover, collinearity analysis revealed 22 and 32 homologous gene pairs between *HpHsf* genes and those of *A. thaliana* and *C. sativus*, respectively (**Figure 3B**). These conserved syntenic relationships highlight the presence of shared ancestral genes and provide valuable clues for inferring the functional roles of *HpHsf* genes.

Plant Heat Shock Factors (HSFs) have a modular structure, typically consisting of several functional domains (Guo et al., 2016). Here, our detailed characterization of canonical Hsf motifs reveals a distinct modular architecture that underscores the functional divergence among the three phylogenetic classes (**Table 1**). The comprehensive suite of motifs in Class A proteins, including the critical AHA activation domain, is consistent with their established role as potent transcriptional activators of the heat stress response (Döring et al., 2000). Notably, HpHsf1 and HpHsf19 lack a canonical NLS and NES, respectively. Given that a defective NES causes nuclear retention in SlHsfA2 (Heerklotz et al., 2001), the absence of these signals in HpHsf1 and HpHsf19 suggests their subcellular localization may be regulated by alternative mechanisms. In Class B, the RD repressor motif (-LFGV-) is conserved at the C-terminus but is notably absent in HpHsf6 and HpHsf13. Phylogenetic analysis placed HpHsf6 in the HsfB5 subfamily (**Figure 2**). This subfamily includes members like StHsfB5 from potato, which lack the RD motif and act as coactivators in heat stress responses (Zhu et al., 2023). Given this and the close evolutionary relationship between HpHsf6 and HpHsf13 (**Figure 3**), both are predicted to function as transcriptional activators regulating stress-responsive genes. However, this prediction requires experimental validation.

Analysis of the motif composition, domain architecture, and gene structure revealed that members within the same subfamily exhibit highly conserved characteristics, indicative of conserved functional roles. The distribution of motifs and conserved domains among HpHsf members follows distinct subfamily-specific patterns. For example, Class A members exclusively contain motifs 6, 7, or 8 and consistently harbor the HSF_DNA-bind domain. In contrast, Class B members are characterized by the presence of motifs 5 or 10, with the exception of *HpHsf15* (**Figure 4**). Similar subfamily-specific patterns have also been reported in pumpkin (Shen and Yuan, 2020), maize (Lin et al., 2011), and tea plant (Li et al., 2025). Furthermore, analysis of cis-acting elements in the promoter regions provided insights into the potential regulatory mechanisms of *HpHsf* genes (Hernandez-Garcia and Finer, 2014). Hsfs are integral components of complex signaling networks that orchestrate plant responses to diverse abiotic stresses, particularly heat (Andrási et al., 2020). They also act as modulators of transcriptional dynamics, fine-tuning stress responses while maintaining a balance between adaptation and normal developmental processes (Bakery et al., 2024). Consistent with these roles, our analysis revealed that *HpHsf* promoters are enriched in cis-acting elements associated not only with stress responses, but also with hormone signaling and developmental processes (**Figure 5**). Research has shown that red and blue light regulate *Hsf* gene expression in species such as medicinal plant *Astragalus mongholicus* and *Cannabis sativa* L., with corresponding effects on cannabinoids accumulation. In addition, light-responsive cis-elements have been identified in the promoters of these *Hsf* genes (Wang et al., 2024; Qian et al., 2023). Similarly, promoters of all *Hphsfs* in *H. pedunculosum* also contain such light-responsive elements, suggesting that their expression may likewise be regulated by light. It is noteworthy that alpine species such as *H. pedunculosum* are routinely exposed to concurrent low-temperature and high-light stress. Our cis-regulatory element analysis revealed that the *HpHsf* genes not only harbor light-responsive elements but also contain low-temperature response elements in seven members (33%). In contrast, while six (26%) of the cucumber *CsHsfs* possess low-temperature responsive elements, no notable distribution of light-responsive elements has been reported in their promoters (Chen et al., 2021). The prevalence of both element types in *H. pedunculosum* suggests a potential coordinated regulatory mechanism for adapting to the complex high-altitude environment. However, these findings are currently based on in silico predictions, and the functional role of these cis-elements requires experimental validation. These findings underscore the multifaceted regulatory roles of *HpHsf* genes in integrating environmental cues with endogenous developmental programs.

High temperatures represent a significant threat to the survival of the alpine plant *H. pedunculosum*. To elucidate the roles of *HpHsf* genes under heat stress, we examined the transcriptional expression profiles of all family members following exposure to 42 °C stress at multiple time points. Two Class A genes (*HpHsf3* and *HpHsf17*) and two Class B genes (*HpHsf2* and *HpHsf5*) were rapidly upregulated within 1 hour of treatment, followed by a noticeable decline by 3 hours (**Figure 6**). Notably, the two Class A members showed further induction at 12 h, suggesting that multiple *Hsf* genes contribute to thermoregulation, with Class A members potentially mediating multi-phase regulatory functions. Interestingly, homologs of *HpHsf3* and *HpHsf17* in cucumber (*CsHsf23* and *CsHsf7*, respectively) also demonstrated rapid responses to high-temperature stress (Chen et al., 2021), implying that these members may have conserved regulatory functions across cucurbitaceous species. Furthermore, the nuclear localization of *HpHsf3, HpHsf5*, and *HpHsf17* provides cytological evidence supporting their roles as transcription factors (**Figure 7**).

## 5 Conclusion

In summary, this study identified 21 *Hsf* genes in the *H. pedunculosum* genome through comprehensive genomic analysis. These genes were systematically characterized by their protein physicochemical properties, chromosomal locations, phylogenetic relationships, conserved motifs, exon-intron structures, protein domains, and cis-regulatory elements. Moreover, we investigated the expression patterns of all *HpHsf* genes under heat stress and confirmed the subcellular localization of three representative HpHsf proteins via transient expression in tobacco epidermal cells. Collectively, these findings offer valuable insights into the *HpHsf* gene family and establish a robust foundation for future functional studies to elucidate their roles in heat stress response.

## Data Availability

The raw genomic dataset of *H. pedunculosum* presented in the study are deposited in the Figshare repository, accession number: https://doi.org/10.6084/m9.figshare.21626153.v2. The original contributions presented in the study are included in the article/**Supplementary Material**, further inquiries can be directed to the corresponding author.
