# Supplementary material for: Genome-wide characterization of the HpHsf gene family and expression analysis under heat stress in Herpetospermum pedunculosum
Source: Front Plant Sci. 2025 Dec 18;16:1701203. doi: 10.3389/fpls.2025.1701203 (PMC12757015; doi:10.3389/fpls.2025.1701203)
Supplement: Supplementary file 1 [file DataSheet1.docx]

Supplementary Material

**
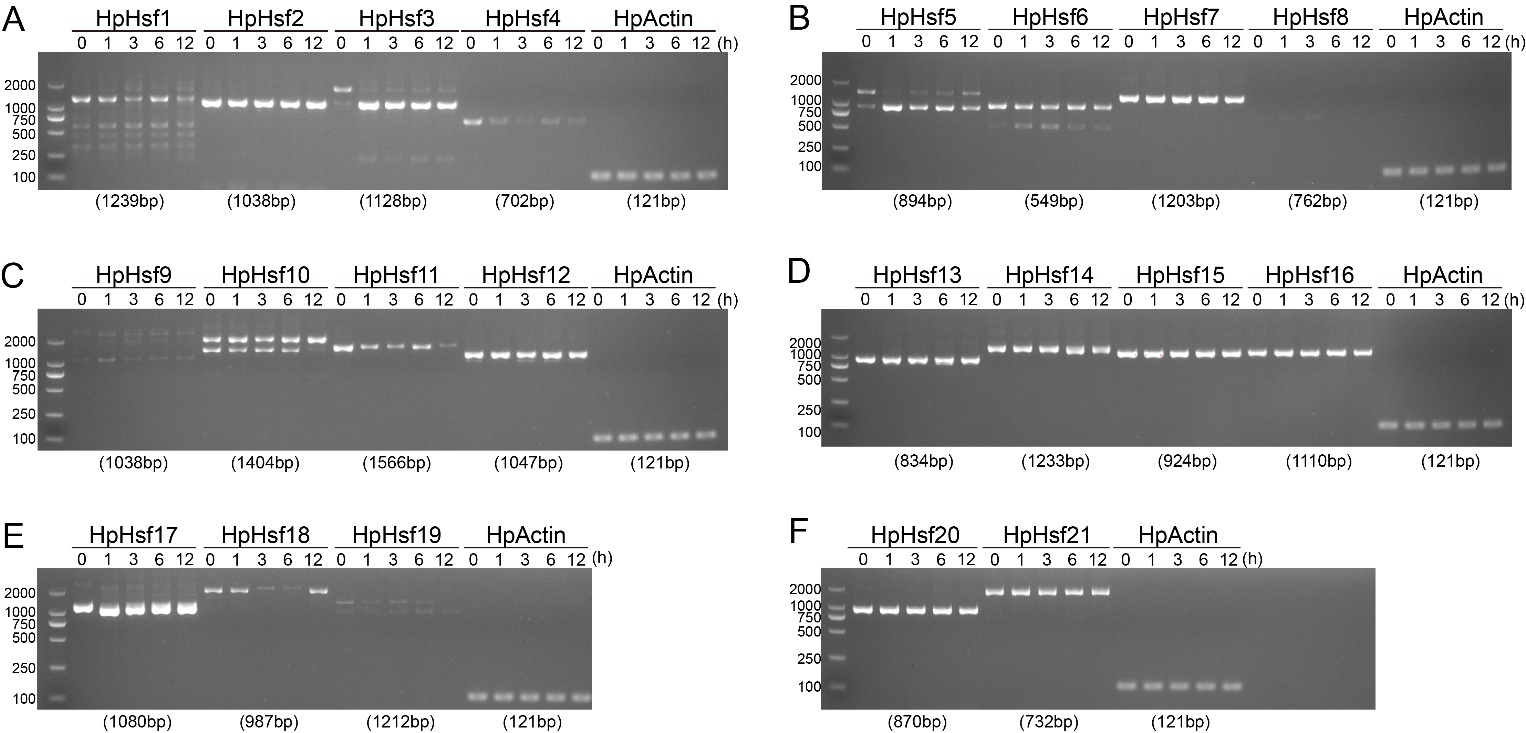
**

**Supplementary Figure 1.** Analysis of *HpHsfs* alternative splicing under heat stress by RT-PCR. (A-F) RT-PCR analysis of 21 *HpHsfs* transcript splicing in *H. pedunculosum* leaves assessed responses to heat stress (42 °C) at various time points (0 h, 1 h, 3 h, 6 h, 12 h). The number at the bottom of each gel image indicates the full-length transcript size for each gene.
